# Mobile Health Interventions to Support Patients With Dementia During the COVID‐19 Pandemic: A Systematic Review

**DOI:** 10.1002/hsr2.72883

**Published:** 2026-07-25

**Authors:** Parastoo Amiri, Neda Malek Mohammadi, Mohammad Sharifi, Ali Mohammadi, Hamid Sharifi, Zahra Galavi, Pooria Afsharifard

**Affiliations:** ^1^ Department of Health Information Technology, School of Allied Medical Sciences Lorestan University of Medical Sciences Khorramabad Iran; ^2^ HIV/STI Surveillance Research Center, and WHO Collaborating Center for HIV Surveillance, Institute for Futures Studies in Health Kerman University of Medical Sciences Kerman Iran; ^3^ Department of Health Information Technology, Faculty of Paramedical Sciences Kermanshah University of Medical Sciences Kermanshah Iran; ^4^ Department of Health Information Technology, School of Allied Medical Sciences Zabol University of Medical Sciences Zabol Iran; ^5^ Department of Health Information Management School of Health Management and Information Sciences, Iran University of Medical Sciences Tehran Iran

**Keywords:** COVID‐19, dementia, mobile health, systematic review, telemedicine

## Abstract

**Background and Aims:**

During the COVID‐19 pandemic, mobile health (mHealth) utilization increased, especially among specific groups such as patients with dementia (PwD). This systematic review aimed to assess mHealth interventions among PwD during the COVID‐19 pandemic.

**Methods:**

This systematic review followed the PRISMA 2020 guidelines. PubMed, Scopus, and Web of Science were searched for English‐language studies published between January 2019 and November 2025. Quantitative, qualitative, and mixed‐methods studies reporting mHealth interventions for PwD during the COVID‐19 pandemic were eligible. Study selection and data extraction were independently conducted by three reviewers. A thematic synthesis based on the Unified Theory of Acceptance and Use of Technology (UTAUT) framework was conducted using ATLAS.ti version 8.

**Results:**

Of the 16,691 records identified, 13 studies met the inclusion criteria, including 7 quantitative, 3 qualitative, and 3 mixed‐methods studies conducted across 12 countries. The synthesis identified 36 drivers and 21 barriers influencing the adoption of mHealth interventions among PwD. Key drivers included perceived usefulness, ease of use, enhanced caregiver support, improved communication with healthcare professionals, and improved access to care. Major barriers comprised technical limitations, inadequate digital infrastructure, language and accessibility challenges, and limited digital literacy among caregivers.

**Conclusion:**

mHealth interventions represent feasible tools for supporting PwD during public health emergencies by enabling remote monitoring, caregiver support, and continuity of care. In practice, healthcare providers should prioritize user‐friendly mHealth solutions tailored to caregivers' digital literacy and dementia severity. At the policy level, investments in digital infrastructure, technical support, and caregiver training programs are essential to reduce adoption barriers and ensure equitable implementation of mHealth interventions.

## 1 Introduction

Dementia has become one of the most pressing issues in the aging population, and its prevalence is increasing. According to projections, the number of patients with dementia (PwD) is expected to reach 139 million by 2050 [1]. The World Health Organization (WHO) has identified dementia and the need to support for PwD as a key public health concern [2]. This disease not only affects PwD but also their caregivers and families [3]. While pharmacological treatments are available, they are often insufficient to slow disease progression and may be associated with adverse side effects. Consequently, dementia care remains largely supportive [4, 5].

The COVID‐19 pandemic imposed restrictions on the lives of many individuals, including PwD and their relatives [6, 7]. Due to factors such as old age, dementia, and other underlying health conditions, these individuals are at a higher risk of severe complications and hospitalization. Therefore, adhering to social distancing guidelines and staying at home were highly recommended for their safety [8]. However, staying at home and maintaining physical distance have also limited their mobility and social interactions and increased behavioral symptoms [9, 10]. Various technologies are available to support dementia care, including mobile health (mHealth). mHealth refers to the use of mobile and wireless technologies, such as smartphones, tablets, and short message service (SMS), to deliver health‐related services and interventions. mHealth is commonly regarded as a subset of telehealth, which broadly encompasses various forms of remote healthcare delivery, including video consultations and web‐based platforms. In this review, telehealth interventions were considered only when they were delivered via mobile devices, as the focus was on portable and widely accessible technologies that were particularly relevant during the COVID‐19 pandemic [11].

Nonpharmacological interventions have emerged as a way to mitigate the challenges posed by the COVID‐19 pandemic. Among these interventions is the use of mHealth applications to support PwD and their caregivers [12]. The rise of mobile technology has led to a digital revolution in healthcare, introducing tools like mHealth, telemedicine, and digital platforms. The WHO defines mHealth as the use of mobile devices and wireless tools for medical and public health purposes. The COVID‐19 pandemic greatly accelerated this shift, making the adoption of remote mHealth services essential and widespread. Furthermore, mHealth has been recommended as an educational and supportive intervention for caregivers of PwD [13, 14]. Various mHealth‐based assistive technologies for dementia care have been proposed, including (1) daily life‐based cognitive training activities, (2) monitoring, (3) dementia screening, (4) reminiscence and socialization, (5) follow‐up, and (6) caregiver support [15].

The use of information and communication technology, particularly mHealth applications, has been widely researched in the context of dementia during the COVID‐19 pandemic [16, 17, 18]. For example, Lai et al. [17] demonstrated the positive impact of mobile video conferencing on the quality of life (QoL) of older adults with neurocognitive disorders during the pandemic. Other studies have indicated that mHealth applications can support the care of older adults with dementia by potentially reducing social isolation, improving memory, monitoring health conditions, and enabling remote communication [19]. These applications can be customized to meet individual needs and preferences, providing a cost‐effective solution for healthcare organizations. While several systematic reviews have explored the use of various technologies for dementia care [20, 21, 22, 23, 24, 25, 26], no comprehensive systematic review has specifically investigated mHealth interventions designed to support PwD during the COVID‐19 pandemic. Other systematic reviews have explored interventions such as telemedicine and telerehabilitation for dementia care during COVID‐19 [27, 28]. The aim of this review was to investigate mHealth interventions supporting PwD during the pandemic and to identify the drivers and barriers to implementing these interventions.

## 2 Methods

We followed the Preferred Reporting Items for Systematic Reviews and Meta‐Analyses (PRISMA) guidelines to report the findings of this review [29].

### 2.1 Data Sources and Search Strategy

We conducted a search of several databases, including PubMed/MEDLINE, Scopus, and Web of Science (Clarivate Analytics), for primary studies published in English from January 2019 to November 2025. Table 1 presents the search strategy. We identified keywords through a review of the Medical Subject Headings (MeSH) system, free‐text terms, expert opinions, and relevant primary studies and reviews. Our search utilized two categories of keywords: those related to mHealth (e.g., “Mobile” OR “Mobile‐phone” OR “M‐health”) and those related to dementia (e.g., “Dementia” OR “Alzheimer” OR “Frontotemporal Dementia”).

**Table 1:** Groups of keywords used in the search strategy.

PubMed	((“E‐health”[Title/Abstract] OR “Ehealth”[Title/Abstract] OR “Mobile”[Title/Abstract] OR “Mobile‐phone”[Title/Abstract] OR “M‐health”[Title/Abstract] OR “mHealth”[Title/Abstract] OR “Mobile‐health”[Title/Abstract] OR “Mobile healthcare”[Title/Abstract] OR “Cell‐phone”[Title/Abstract] OR “Cell phone”[Title/Abstract] OR “Smart phone”[Title/Abstract] OR “Smartphone”[Title/Abstract] OR “Smart‐phone”[Title/Abstract] OR “Handheld”[Title/Abstract] OR “Tablet”[Title/Abstract] OR “Mobile tablet”[Title/Abstract] OR “App”[Title/Abstract] OR “Mobile‐app”[Title/Abstract] OR “Mobile telephone”[Title/Abstract] OR “Mobile app”[Title/Abstract] OR “Mobile app in healthcare”[Title/Abstract] OR “mHealth app”[Title/Abstract] OR “Mobile healthcare app”[Title/Abstract] OR “Short Message service”[Title/Abstract] OR “SMS”[Title/Abstract] OR “Text‐message”[Title/Abstract] OR “Text message”[Title/Abstract] OR “Text messaging”[Title/Abstract] OR “Message reminder”[Title/Abstract] OR “Voice‐message”[Title/Abstract] OR “Voice message”[Title/Abstract]) AND (“Dementi*”[Title/Abstract] OR “Alzheimer*”[Title/Abstract] OR “Frontotemporal Dementia”[Title/Abstract] OR “Vascular Dementia”[Title/Abstract] OR “Lewy body”[Title/Abstract] OR “*Cognitive decline”[Title/Abstract] OR “Cognitive disorder”[Title/Abstract] OR “Cognitive impairment”[Title/Abstract] OR “Multi‐Infarct Dementia”[Title/Abstract])) AND (“COVID‐19”[Title/Abstract] OR “covid19”[Title/Abstract] OR “2019 novel coronavirus”[Title/Abstract] OR “2019 nCoV”[Title/Abstract] OR “SARS‐CoV‐2”[Title/Abstract])
Scopus	(TITLE‐ABS‐KEY (“E‐health” OR “Ehealth” OR “Mobile” OR “Mobile‐phone” OR “M‐health” OR “mHealth” OR “Mobile‐health” OR “Mobile healthcare” OR “Cell‐phone” OR “Cell phone” OR “Smart phone” OR “Smartphone” OR “Smart‐phone” OR “Handheld” OR “Tablet” OR “Mobile tablet” OR “App” OR “Mobile‐app” OR “Mobile telephone” OR “Mobile app” OR “Mobile app in healthcare” OR “mHealth app” OR “Mobile healthcare app” OR “Short Message service” OR “SMS” OR “Text‐message” OR “Text message” OR “Text messaging” OR “Message reminder” OR “Voice‐message” OR “Voice message”) AND TITLE‐ABS‐KEY (“Dementi*” OR “Alzheimer*” OR “Frontotemporal Dementia” OR “Vascular Dementia” OR “Lewy body” OR “*Cognitive decline” OR “Cognitive disorder” OR “Cognitive impairment” OR “Multi‐Infarct Dementia”) AND TITLE‐ABS‐KEY (“COVID‐19” OR “covid19” OR “2019 novel coronavirus” OR “2019 nCoV” OR “SARS‐CoV‐2”))
Web of Science	“E‐health” OR “Ehealth” OR “Mobile” OR “Mobile‐phone” OR “M‐health” OR “mHealth” OR “Mobile‐health” OR “Mobile healthcare” OR “Cell‐phone” OR “Cell phone” OR “Smart phone” OR “Smartphone” OR “Smart‐phone” OR “Handheld” OR “Tablet” OR “Mobile tablet” OR “App” OR “Mobile‐app” OR “Mobile telephone” OR “Mobile app” OR “Mobile app in healthcare” OR “mHealth app” OR “Mobile healthcare app” OR “Short Message service” OR “SMS” OR “Text‐message” OR “Text message” OR “Text messaging” OR “Message reminder” OR “Voice‐message” OR “Voice message” (Topic) AND “Dementi*” OR “Alzheimer*” OR “Frontotemporal Dementia” OR “Vascular Dementia” OR “Lewy body” OR “*Cognitive decline” OR “Cognitive disorder” OR “Cognitive impairment” OR “Multi‐Infarct Dementia” (Topic) AND “COVID‐19” OR “covid19” OR “2019 novel coronavirus” OR “2019 nCoV” OR “SARS‐CoV‐2” (Topic)

### 2.2 Eligibility Criteria

The inclusion criteria in this study were as follows: (1) the interventions were delivered through a mobile device (e.g., mobile phone, Personal Digital Assistant), (2) the target population was PwD, (3) the study was conducted during the COVID‐19 pandemic, and (4) the study was an original research article. The exclusion criteria included studies whose objectives were outside the scope of this review, review articles, opinion papers, editorials, letters to the editor, protocols, conferences abstracts, and books.

### 2.3 Study Selection

We imported all retrieved records into Mendeley Reference Manager [30]. We then removed duplicate records, and uploaded the remaining records to Rayyan for screening [31]. In the screening stage, the titles and abstracts of all articles were reviewed independently by all three authors (P. A., Z. G., and N. M. M.) based on the inclusion and exclusion criteria. The full texts of potentially eligible studies were independently assessed by the three reviewers. The reference lists of the included studies were also screened to identify additional relevant studies. Following full‐text assessment, the primary studies that addressed the eligibility criteria were included. In addition, disagreements were resolved based on discussion and agreement between the three authors. In case of disagreement, we used the opinion of the fourth expert in the research team.

### 2.4 Data Extraction and Analysis

We created a data extraction form in Microsoft Word 2016 containing the following information from each study: author(s), publication year, country, type of study, study setting, sample population, study duration, technology intervention, tool type, technology purpose, study purpose, outcomes, drivers, and barriers.

Technology purpose was categorized based on the classification of Mancioppi et al. [32]. Furthermore, mHealth interventions were classified according to the WHO framework into the following categories: communication between individuals and health services, communication between health services and individuals, consultation between healthcare professionals, intersectoral communication in emergencies, health monitoring and surveillance, and access to information for healthcare professionals at the point of care [11, 33].

Three researchers (P. A., Z. G., and N. M. M.) used thematic analysis in ATLAS.ti8 software to identify and extract the drivers and barriers affecting the use of mHealth interventions [34]. All reported facilitators and barriers were extracted verbatim from the included studies, coded independently by three reviewers, grouped into themes and subthemes, and mapped to the UTAUT framework. Multiple drivers and barriers could be identified within a single study; therefore, the reported counts represent unique coded factors rather than the number of studies. Thematic analysis is a qualitative data analysis method that involves examining a data set to identify recurring patterns and extract core themes from the data. The Unified Theory of Acceptance and Use of Technology (UTAUT) combines various theories—grounded in the Technology Acceptance Model (TAM)—to explain users' intentions and behavior in using technology [35]. This theory has been used to evaluate the adoption and use of technology in various areas of mobile and information technology and was used as a theoretical framework for interpreting the findings [36, 37].

### 2.5 Study Quality

Given the substantial heterogeneity in study designs, interventions, and outcome measures, a formal risk‐of‐bias assessment was not feasible. Nevertheless, methodological limitations of the included studies were considered during the interpretation and synthesis of findings.

## 3 Results

**Figure 1:**
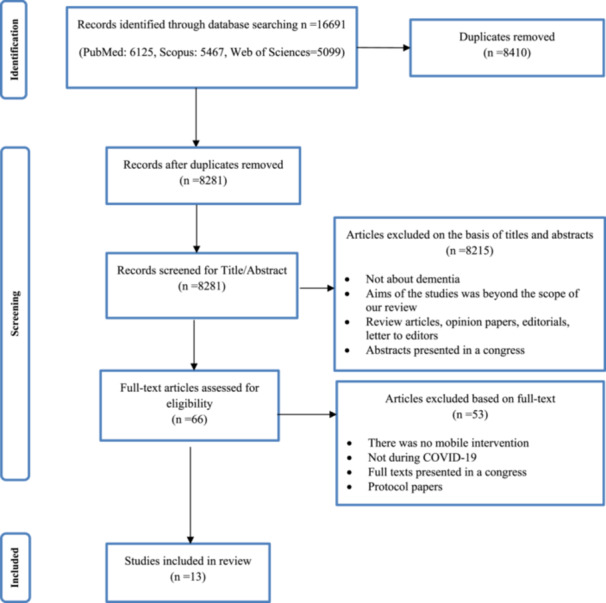
PRISMA flow diagram.

We identified 16,691 articles, including 6125 articles from PubMed, 5467 articles from Scopus, and 5099 articles from Web of Science. After the removal of duplicates, 8281 articles remained for title and abstract screening. Following title and abstract screening, the full text of 66 articles were assessed for eligibility. Finally, 13 articles were included in the final review (Figure 1).

Most of the included studies were published in 2022 (*n* = 10), and all studies, except for the study by Requena‐Komuro et al. [38], focused on assistive technology (Table 2).

**Table 2:** Characteristics of the included studies.

Authors (publication year)	Country	Type of study	Study setting	Sample population	Study duration	Technology intervention	Tool type	Technology Purpose*
Miura et al. [39] (2025)	Japan	Quantitative/RCT study	Nonacademic	Older adults with subjective cognitive concerns (*n* = 75)	3 months	App	Premade/standalone	Assistance
Dorell et al. [40] (2022)	Sweden	Qualitative/description	Nonacademic	Caregivers (*n* = 12)	2 months	App	Premade/standalone	Assistance
Kagwa et al. [41] (2022)	Sweden	Qualitative/description	Nonacademic	Caregivers (*n* = 12)	6 months	App	Premade/standalone	Assistance
Elvas et al. [42] (2022)	Portugal	Quantitative/development study	Academic	PwD (*n* = 15)	3 weeks	Dashboards	Researcher made/standalone	Assistance
Engelsma et al. [43] (2022)	Netherlands	Quantitative/Delphi study	Academic	Case managers, informal caregivers, hospital healthcare professionals, district nurses, and researcher (*n* = 37)	3 months	App	Premade/standalone	Assistance
Requena‐Komuro et al. [38] (2022)	UK	Quantitative/cross‐sectional study	Academic	PwD (*n* = 25)	7 months	Zoom app	Premade/standalone	Treatment
Ottaviani et al. [44] (2022)	Brazil	Mixed methods	Academic	PwD in Stage 1 (*n* = 9), in Stage 2 (*n* = 10)	3 weeks	Online program (iSupport‐Brasil)	Premade/standalone	Assistance
Varshney et al. [45] (2022)	USA	Qualitative/development study	Nonacademic	Older adults, friends, family, and caregivers (*n* = 100)	N/M	App	Researcher made/standalone	Assistance
Ahmed et al. [46] (2022)	Germany & Italy & Portugal & Romania & Spain	Mixed method	Academic	PwD, caregivers, healthcare professionals, key stakeholders (*n* = 217)	4 months	ICT‐based healthcare platform	Premade/standalone	Assistance
Perales‐Puchalt et al. [47] (2022)	USA	Mixed method/cevelopment and usability Study	Nonacademic	Caregivers (*n* = 5)	6 months	SMS	Researcher made/standalone	Assistance
Barisch‐Fritz et al. [48] (2022)	Germany	Quantitative/RCT study	Academic	Nursing assistants (*n* = 10) and individuals with primary dementia (*n* = 13)	18 weeks	InCoPE app	Researcher made/standalone	Assistance
Faieta et al. [49] (2021)	Canada	Quantitative/cross‐sectional study	Nonacademic	Caregivers (*n* = 82)	5 months	App	Premade/standalone	Assistance
Engelsma et al. [19] (2021)	Netherlands	Quantitative/Delphi study	Academic	Case manager, nurse, geriatrician, researcher, informal caregivers (*n* = 44)	N/M	App	Premade/standalone	Assistance

Abbreviations: UK, the United Kingdom; USA, the United States of America; caregivers, caregivers; QoL, quality of life; PwD, people with dementia; N/M, not mentioned; SMS, short message service; ICT, information and communication technology.

* Technology purpose is based on a review by Mancioppi et al. [32].

Two studies were conducted in Sweden [40, 41], two studies in the Netherlands [19, 43], two studies in the USA [45, 47], one study in Portugal [42], one study in the UK [38], one study in Brazil [44], one study in Germany [48], one study in Canada [49], one study in Japan [39], and one study was conducted across several countries (Spain, Germany, Italy, Portugal, and Romania) [46]. Among these, seven articles were quantitative [19, 38, 39, 42, 43, 48, 49], three articles were qualitative [40, 41, 45], and three articles [44, 46, 47] used a mixed‐methods design.

Seven studies were conducted in an academic setting [19, 38, 42, 43, 44, 46, 48], whereas six were conducted in a nonacademic setting [39, 40, 41, 45, 47, 49]. Four studies [40, 41, 47, 49] focused on caregivers, three studies [38, 42, 44] involved PwD, and the remaining studies [19, 39, 43, 45, 46, 48] included a combination of caregivers, patients, and healthcare providers. Study duration ranged from 3 weeks to 7 months; however, two studies [19, 45] did not report this information.

### 3.1 Frequency of Technology Intervention

Table 3 presents the frequency of technology interventions reported in the included studies. Mobile applications (apps) and premade or independent tools were the most frequently reported, each appearing in nine studies [19, 38, 39, 40, 41, 43, 45, 48, 49]. Other interventions included dashboards [42], an online application (iSupport‐Brasil) [44], a platform [46], and SMS [47], each of which was reported in one study.

**Table 3:** Frequency of technology intervention.

Technology intervention	Number of studies	References
Mobile application (App)	9	[19, 38, 39, 40, 41, 43, 45, 48, 49]
Dashboard	1	[42]
Online application	1	[44]
Platform	1	[46]
SMS	1	[47]
Premade/independent tools	9	[19, 38, 39, 40, 41, 43, 45, 48, 49]

### 3.2 Study Objectives and WHO‐Based Classification of mHealth Interventions

The included studies addressed a variety of objectives (Table 4). Three studies aimed to “provide professional support for family caregivers of PwD” [40, 41, 47], and three studies focused on “determining barriers, requirements, and underlying factors to using mHealth for PwD” [19, 43, 46]. Three studies examined “effectiveness” [39, 48, 49], two studies explored “feasibility” [38, 47], and two studies investigated “acceptability” [41, 47]. Because individual studies could address multiple objectives, the total frequency exceeds the number of included studies. According to the WHO framework, the application focus of the identified mHealth interventions was classified into three categories: “communication between health services and individuals” [38, 39, 40, 41, 47, 48, 49], “access to information for healthcare professionals at the point of care” [42], and “health monitoring and surveillance” [45].

**Table 4:** Study objectives and WHO‐based application focus of mHealth interventions for PwD during the COVID‐19 pandemic.

*Objectives*
Providing professional support for family caregivers of PwD [40, 41, 47]
Reducing pressure on busy staff and avoid all unnecessary visits to hospital facilities by mHealth [42]
Determining barriers, requirements, and underlying factors to using mHealth for PwD [19, 43, 46]
Adapting neuropsychological tests for online administration [38]
Assessing usability and acceptability of mHealth to support family caregivers of PwD [44]
Focusing on a mHealth intervention for PwD [45]
Effectiveness [39, 48, 49]
Feasibility [38, 47]
Acceptability [41, 47]
Efficiency [43]
*Application focus*
Communication between health services and individuals [38, 39, 40, 41, 47, 48, 49]
Access to information for healthcare professionals at point of care [42]
Health monitoring and surveillance [45]

Abbreviations: PwD, people with dementia; mHealth, mobile health.

### 3.3 Outcomes of Included Studies

The reported outcomes of mHealth interventions were categorized as positive or negative. Overall, more positive outcomes (*n* = 8) than negative outcomes (*n* = 5) were identified. Table 5 summarizes the outcomes reported in the included studies. Of the 13 included studies, 10 reported outcome‐related findings and were therefore included in Table 5, whereas the remaining three studies did not provide outcome information.

**Table 5:** Positive and negative outcomes of mHealth interventions among PwD during the COVID‐19 pandemic.

Positive outcomes	Negative outcomes
Enhanced caregiver support and improved communication with healthcare professionals [40, 42, 48]	Increased psychological distress and adverse mental health symptoms among users [49]
Reduced caregiver workload and care‐management burden [42]	Increased anxiety among PwD during periods of caregiver separation [49]
Improved access to healthcare services and continuity of care during pandemic restrictions [45]	Increased behavioral symptoms, including restlessness and agitation, among PwD [43]
Maintained caregiver–provider communication and care delivery despite reduced in‐person visits [39, 40]	Reduced QoL among PwD and/or caregivers [49]
Evaluation of factors influencing successful implementation of mHealth interventions [19]	Reduced social engagement and interpersonal interaction [39]
Identification of user‐related factors affecting mHealth adoption and engagement [46]	
Assessment of user perceptions and determinants of mHealth acceptance and use [44]	
Improved coordination of care activities and adherence to daily care routines through digital support tools [43]	

Abbreviations: PwD, people with dementia; mHealth, mobile health; QoL, quality of life.

The most frequently reported positive outcome was enhanced caregiver support and improved communication with healthcare professionals, which was identified in three studies [40, 42, 48]. Other positive outcomes included reduced caregiver workload and care‐management burden [42], improved access to healthcare services and continuity of care during pandemic restrictions [45], maintained caregiver–provider communication and care delivery despite reduced in‐person visits [39, 40], evaluation of factors influencing the successful implementation of mHealth interventions [19], identification of user‐related factors affecting mHealth adoption and engagement [46], assessment of user perceptions and determinants of mHealth acceptance and use [44], and improved coordination of care activities and adherence to daily care routines through digital support tools [43].

Negative outcomes were reported in three studies [39, 43, 49]. These included increased psychological distress and adverse mental health symptoms among users [49], increased anxiety among PwD during periods of caregiver separation [49], reduced QoL among PwD and/or caregivers [49], increased behavioral symptoms such as restlessness and agitation among PwD [43], and reduced social engagement and interpersonal interaction [39].

### 3.4 Drivers and Barriers Based on the UTAUT Model

Numerous drivers (*n* = 36) and barriers (*n* = 21) were extracted from the included studies. Based on the UTAUT framework, the identified drivers were categorized into performance expectancy, effort expectancy, social influence, and facilitating conditions. Barriers were analyzed separately and grouped into infrastructural and personal barriers according to their characteristics. In our study, we have reported these barriers under the section “Barriers and problems,” further organizing them into two main subcategories: infrastructural and personal barriers, to better reflect the types of barriers identified in the reviewed studies.

### 3.5 Performance Expectancy

Reported drivers included cost savings [19, 45], improved communication between PwD with caregivers [46], enhanced care coordination [46], and improved QoL of patients and caregivers [45, 48]. Six studies reported involving end‐users (patients [38, 49], caregivers [40, 41, 44, 47, 49], healthcare providers [38], and nursing homes [48]) in the design, selection, and implementation of mHealth interventions. Reported social drivers included reduced social isolation, improved communication with family members and healthcare providers, and caregiver education and skills training [19, 44].

### 3.6 Effort Expectancy

Out of the 13 included studies, 3 explicitly reported findings related to effort expectancy. For instance, one study [40] mentioned capabilities such as chat functionality, web links to relevant sites, and a personalized contact list. Another study [44] utilized in health interventions with minimal technical complexity designed for early‐stage dementia users. In a study [19], researchers emphasized that mHealth interventions for the early stages of dementia were designed to minimize technological complexity for users, for example, by simplifying navigation, reducing the number of steps required to perform tasks, and providing reminders to support adherence.

### 3.7 Barriers and Problems

Included studies identified a range of barriers to the adoption and use of mHealth interventions among PwD and their caregivers. These barriers were grouped into two categories: infrastructural barriers and personal barriers. A summary of the identified barriers is presented in Table 6.

**Table 6:** Barriers to mHealth adoption among PwD during the COVID‐19 pandemic.

Barrier	Category	References
Need for internet access	Infrastructural	[38, 40, 41, 45]
Need for specific software	Infrastructural	[38, 42]
Dependence on wireless networks for video upload/download	Infrastructural	[45]
Scalability challenges	Infrastructural	[45]
Organizational pressure	Infrastructural	[19]
Limited use in home care settings	Infrastructural	[19]
Infrastructure challenges	Infrastructural	[45]
Privacy and data protection concerns	Infrastructural	[46]
Complex setup and maintenance requirements (e.g., charging and updating devices)	Infrastructural	[19]
Language limitations	Personal	[40, 41, 46, 47, 48, 49]
Need for hardware (computer, tablet, smartphone)	Personal	[38, 40, 41, 44, 45, 48, 49]
Need for email and social media access	Personal	[49]
Hearing or visual requirements	Personal	[38]
Unmet needs related to daily activities and health management	Personal	[19]
Lack of perceived need for mHealth interventions	Personal	[19]
Limited suitability for advanced dementia	Personal	[19]
Frustration with complex technologies or tasks	Personal	[19]
Lack of awareness of available interventions	Personal	[19]
Resistance to technology use	Personal	[19]
Additional burden on caregivers	Personal	[19]
Physical requirements (e.g., ability to walk 10 meters)	Personal	[49]

### 3.8 Infrastructural Barriers

Infrastructural barriers included the need for internet access [38, 40, 41, 45], specific software requirements [38, 42], dependence on wireless networks for video uploading and downloading [45], scalability challenges [45], organizational pressure [19], limited applicability in home‐care settings [19], infrastructure‐related limitations [45], privacy and data protection concerns [46], and intervention setup and maintenance requirements, such as charging devices and performing software updates [19].

### 3.9 Personal Barriers

Personal barriers included language limitations [40, 41, 46, 47, 48, 49], the need for hardware devices such as computers, tablets, or smartphones [38, 40, 41, 44, 45, 48, 49], the need for email and social media accounts [49], lack of awareness of available interventions [19], lack of perceived need for mHealth interventions [19], resistance to technology use [19], and the additional burden placed on caregivers [19]. One study suggested that mHealth interventions may have limited suitability for individuals with advanced dementia [19]. Other barriers included sensory limitations, such as hearing or visual impairments, that could affect the use of digital interventions [38].

## 4 Discussion

This systematic review identified a variety of mHealth intervention, including mobile applications, dashboards, platforms, SMS services, and online programs, used to support PwD during the COVID‐19 pandemic. Most studies aimed to provide professional support to family members and caregivers of PwD [40, 41, 47]. Among the reported outcomes, the most frequently identified positive outcome was enhanced caregiver support and improved communication with healthcare professionals [40, 42, 48]. Support for caregivers included guidance on caregiving activities, patient status monitoring, and strategies to reduce caregiver burden. In contrast, support for healthcare professionals primarily involved facilitating communication with patients and caregivers, enabling remote monitoring, and improving care coordination. These findings suggest that mHealth interventions played an important role in strengthening support systems for both caregivers and healthcare professionals during the pandemic.

However, several negative outcomes were also reported, including increased psychological distress and adverse mental health symptoms among users, increased anxiety among PwD during periods of separation from their caregivers, reduced QoL [49], increased restlessness and agitation [43], and the complexity associated with using mHealth interventions [43]. Identified barriers to the use of mHealth interventions among PwD were reported as technical barriers in approximately 16% of studies, infrastructural barriers in 50%, and personal barriers in 83% of studies. From the perspective of performance expectancy, the primary benefits of mHealth interventions included improvements in QoL, enhanced caregiver support, and better care coordination.

The primary objective of the studies was to support family members and caregivers of PwD. This is consistent with previous research that identifies family members' and caregivers' support as a key area for the use of mHealth in dementia care [14, 15]. Caring for a PwD can be a complex and challenging task that places a significant burden on caregivers. Caregivers of PwD are at risk of experiencing negative outcomes, such as stress, anxiety, burden, and low QoL, especially in end‐stage dementia [50]. Therefore, addressing the care burden of caregivers and providing them with high levels of support is a priority [51]. Support can come in the form of social groups, education and training, information services, respite care, case management approaches, and psychosocial help [52]. These services aim to alleviate caregiver stress, enhance their knowledge and skills, provide much‐needed breaks through respite care, and offer emotional support. By supporting family members and caregivers of PwD, mHealth interventions can improve the overall caregiving experience and outcomes.

The outcomes of the mHealth interventions examined in this study focused on providing uninterrupted support to PwD and their caregivers, improving treatment outcomes, and reducing the burden associated with caregiving. These interventions ensure that PwD and caregivers could receive assistance and guidance whenever needed, even outside traditional healthcare settings [53]. During the pandemic, mHealth interventions provided uninterrupted remote support, which was critical when in‐person visits were restricted. Previous studies have reported that mHealth interventions can improve clinical outcomes, disease management, and adherence to treatment and care [54, 55]. These interventions can include medication reminders, symptom tracking, and personalized care plans, promoting treatment adherence and better disease management [56]. However, it is important to note that while mHealth interventions show promise, they should be considered complementary tools to traditional healthcare approaches.

Despite the potential positive outcomes, it is important to acknowledge that mHealth interventions may also have negative outcomes. Negative outcomes included heightened anxiety and restlessness, as well as a decline in QoL for both PwD and their caregivers, indicating that mHealth interventions are not always favorably received. These negative outcomes suggest that while mHealth interventions have the potential to be helpful, they may not always be well‐received or suitable for every individual. This is consistent with the findings of Engelsma et al. [19], which showed that older adults PwD may be resistant to mHealth due to anxiety or suspicion, as this technology may be unfamiliar to them. This resistance and anxiety may stem from a lack of understanding or comfort with digital devices, or concerns about privacy and data security.

Furthermore, our study found that infrastructural and personal barriers can hinder the utilization of mHealth by PwD and their caregivers. Rathnayake et al. [14] categorized these barriers into three categories: technology, literacy, and time, which aligns with our study's findings on technical barriers. Their study indicated that the lack of experience and skills among caregivers in using mHealth programs is the primary barrier to adoption. Older age appeared to be an important contributing factor intensifying these barriers. Specifically, older caregivers often faced challenges in adopting digital technology due to limited digital literacy (literacy barrier), discomfort with devices (technology barrier), and lower confidence in using apps and platforms, which can also increase the time required to navigate mHealth tools (time barrier). Additionally, O'Connor et al. [57] found that caregivers face challenges in utilizing mobile technology, and negative attitudes toward technology can limit their participation in mHealth program design. These age‐related challenges may impact adherence, engagement, and the effectiveness of interventions, highlighting the need for user‐friendly designs and targeted training for older caregiver populations.

The present study found that performance expectation is one of the main drivers for using mHealth interventions to support PwD. This finding is consistent with several other studies that have reported the main benefit of health interventions as improving the QoL of patients and their caregivers, based on performance expectations [58, 59, 60]. Before the COVID‐19 pandemic, numerous mHealth interventions for PwD were developed to support cognitive assessments, including the Saint Louis University Mental Status Examination, the Standardized Mini‐Mental State Examination, and the Montreal Cognitive Assessment [61, 62]. In the studies included in this review, no interventions specifically focused on cognitive testing were reported; instead, interventions emphasized caregiving support, communication, and disease management.

Smart personal device apps can be useful for dementia caregivers, and many apps offer features that facilitate communication or remote care. Caregivers who are comfortable using innovative personal technology can utilize various remote communication methods, such as messaging, video chat, social media, and email, to stay connected with their loved ones or care center staff. Interventions included in this review which spanned the pandemic emphasized caregiver support, communication, and disease management.

While the present review focused specifically on the COVID‐19 pandemic period, future research should examine mHealth interventions for PwD in the post‐pandemic era. A systematic search covering the years 2023–2025 without the term “COVID‐19” in the search string could provide valuable insights into how mHealth interventions have evolved following the end of the public health emergency.

Beyond its immediate role during the COVID‐19 pandemic, the rapid adoption of mHealth created opportunities for the long‐term transformation of dementia care. The widespread use of remote communication, monitoring, caregiver support, and digital care coordination demonstrated the feasibility of integrating mHealth into routine healthcare services. Lessons learned during the pandemic may guide the development of more user‐centered, accessible, and scalable mHealth solutions for PwD and their caregivers. Future developments should focus on improving usability, digital literacy support, interoperability with healthcare systems, and personalized care delivery to maximize the long‐term benefits of mHealth.

This study has three limitations. First, we only included articles published in English, which may have led to the exclusion of relevant articles published in other languages due to publication bias. Second, we limited the scope of the research to three databases, PubMed, Scopus, and Web of Science, which could result in the omission of some qualified and important articles. This issue, we also examined the references of the retrieved articles to ensure that we did not omit any relevant studies. Third limitation of this review is the time frame of the included studies. Since our focus was specifically on the COVID‐19 pandemic period, few relevant studies have been published after 2023. Consequently, the number of citations from 2024 to 2025 is limited, which reflects the natural decline in pandemic‐related research following the end of the public health emergency.

## 5 Conclusion

The COVID‐19 pandemic significantly accelerated the worldwide adoption of mHealth services. For PwD, mHealth interventions proved to be valuable, suitable, and feasible alternatives to face‐to‐face communication, according to the studies reviewed. However, several barriers limited their acceptance and further development, including technical issues (e.g., unreliable internet), infrastructural gaps (e.g., lack of devices or email access), and personal factors (e.g., hesitancy with social media or digital tools).

At the same time, PwD were especially vulnerable to the effects of pandemic lockdowns, which worsened their medical conditions. The pandemic also drove improvements in digital services, leading to more widespread integration of mHealth into healthcare systems. The authors argue that policymakers must address these barriers, as technological interventions can help stakeholders better support PwD. Governments can use knowledge of these barriers to design solutions that improve mHealth utilization among older adults living with dementia.

## Author Contributions

**Parastoo Amiri:** conceptualization, writing – original draft, data curation, methodology, writing – review and editing, formal analysis. **Neda Malek Mohammadi:** data curation, methodology, formal analysis. **Mohammad Sharifi:** data curation, methodology, investigation. **Ali Mohammadi:** investigation, resources, software. **Hamid Sharifi:** investigation, methodology. **Zahra Galavi:** data curation, supervision, methodology, formal analysis, conceptualization, writing – review and editing, writing – original draft. **Pooria Afsharifard:** data curation, investigation, methodology, writing – review and editing, writing – original draft. All authors have read and approved the final version of the manuscript.

## Funding

The authors have nothing to report.

## Ethics Statement

This article reports on an independent research project conducted in the field of medical informatics at Kermanshah University of Medical Sciences (KUMS), without organizational support. The study was approved by the ethics committee of KUMS (code of ethics: IR.KUMS.REC.1402.190) and was conducted in accordance with the ethical guidelines of the Helsinki Declaration. The study was also supported by the Student Research Committee of KUMS (Code: 4020343).

## Consent

The authors have nothing to report.

## Conflicts of Interest

The authors declare no conflicts of interest.

## Transparency Statement

Miss Zahra Galavi is the guarantor. Zahra Galavi affirms that this manuscript is an honest, accurate, and transparent account of the study being reported; that no important aspects of the study have been omitted; and that any discrepancies from the study as planned (and, if relevant, registered) have been explained.

## Data Availability

Our data or material may be available from the corresponding author or first author upon reasonable request. Zahra Galavi had full access to all of the data in this study and takes complete responsibility for the integrity of the data and the accuracy of the data analysis.
